# Copper-to-copper direct bonding on highly (111)-oriented nanotwinned copper in no-vacuum ambient

**DOI:** 10.1038/s41598-018-32280-x

**Published:** 2018-09-17

**Authors:** Jing-Ye Juang, Chia-Ling Lu, Kuan-Ju Chen, Chao-Chang A. Chen, Po-Ning Hsu, Chih Chen, K. N. Tu

**Affiliations:** 1 Department of Materials Science and Engineering, National Chiao Tung University, Hsinchu, 30010 R.O.C Taiwan; 2 Department of Materials Science and Engineering, University of California at Los Angeles, Los Angeles, California 90095 USA; 3 Department of Mechanical Engineering, National Taiwan University of Science and Technology, Taipei, 10607 R.O.C Taiwan

## Abstract

A vacuum-free Cu-to-Cu direct bonding by using (111)-oriented and nanotwinned Cu has been achieved. A fast bonding process occurs in 5 min under a temperature gradient between 450 and 100 °C. It is verified by grain growth across the bonded interface. To investigate the grain growth behavior, further annealing in the temperature gradient, as well as in a reversed temperature gradient, was performed. They showed similar recrystallization behavior with de-twinning. To analyze the de-twinning, we recall the classic model of annealing twin formation by Fullman and Fisher as comparison. Our case is opposite to the model of Fullman and Fisher. A mechanism of direct bonding by surface diffusion creep is proposed.

## Introduction

Cu-to-Cu direct bonding has been studied intensively for years because it has the potential to replace solder joints in advanced packaging technology^1–8^. An example is the application in complementary-metal-oxide-semiconductor image sensors^9,10^. In previous studies, several direct Cu-to-Cu bonding techniques have been investigated. Ultrahigh vacuum (UHV) with radioactive pretreatment of atomically flat surfaces has been reported for room temperature bonding^11–13^. However, ion beam cleaning under UHV condition is high in cost and technically incompatible with mass production processes. Another method was to apply a noble metal to passivate the thin film surfaces. It was applied to a pre-bonded surface below 200 °C, followed by annealing at 400 °C for 60 min^14–16^. Nevertheless, the use of a noble metal is high in cost too. The pre-bonding process followed by annealing is also very time-consuming.

(111) oriented and nanotwinned Cu (nt-Cu) has attracted much attention due to its high surface diffusivity on the (111) plane^17,18^. The rapid surface diffusion has enabled low-temperature direct bonding in the range of 150 to 200 °C under an ordinary vacuum of 10^−4^ to 10^−3^ torr. Many studies on nt-Cu focus on the wafer-to-wafer bonding technique under isothermal and vacuum conditions. Very few studies have been conducted on chip-to-wafer bonding, under a temperature gradient in ambient conditions, without vacuum. Cu-to-Cu bonding with a temperature gradient in ambient conditions can satisfy the industrial demand for heterogeneous chip integration with commercially available bonding equipment. In addition, a well-bonded Cu-to-Cu joint comprises two key steps. First, surface diffusion by creep to achieve bonding of two surfaces. Second, grain growth to eliminate the bonding interface^16–18^. There are several literatures addressing the first step. However, very few efforts have been made to understand the grain growth across the bonding interface.

In this study, we report a vacuum-free Cu-to-Cu direct bonding technique with a short bonding time of 5 min by using (111)-oriented nt-Cu, with no additional annealing. The bonding is verified through the grain growth occurring across the bonding interface. In addition, we found that the direction of grain growth is not dependent on the direction of the applied temperature gradient because of recrystallization. We recall the classic model of annealing twin formation by Fullman and Fisher for comparison. In addition, a surface diffusion creep-assisted mechanism of bonding is proposed.

## Results

### Fabrication of test dies and Cu surface treatment

**Figure 1:**
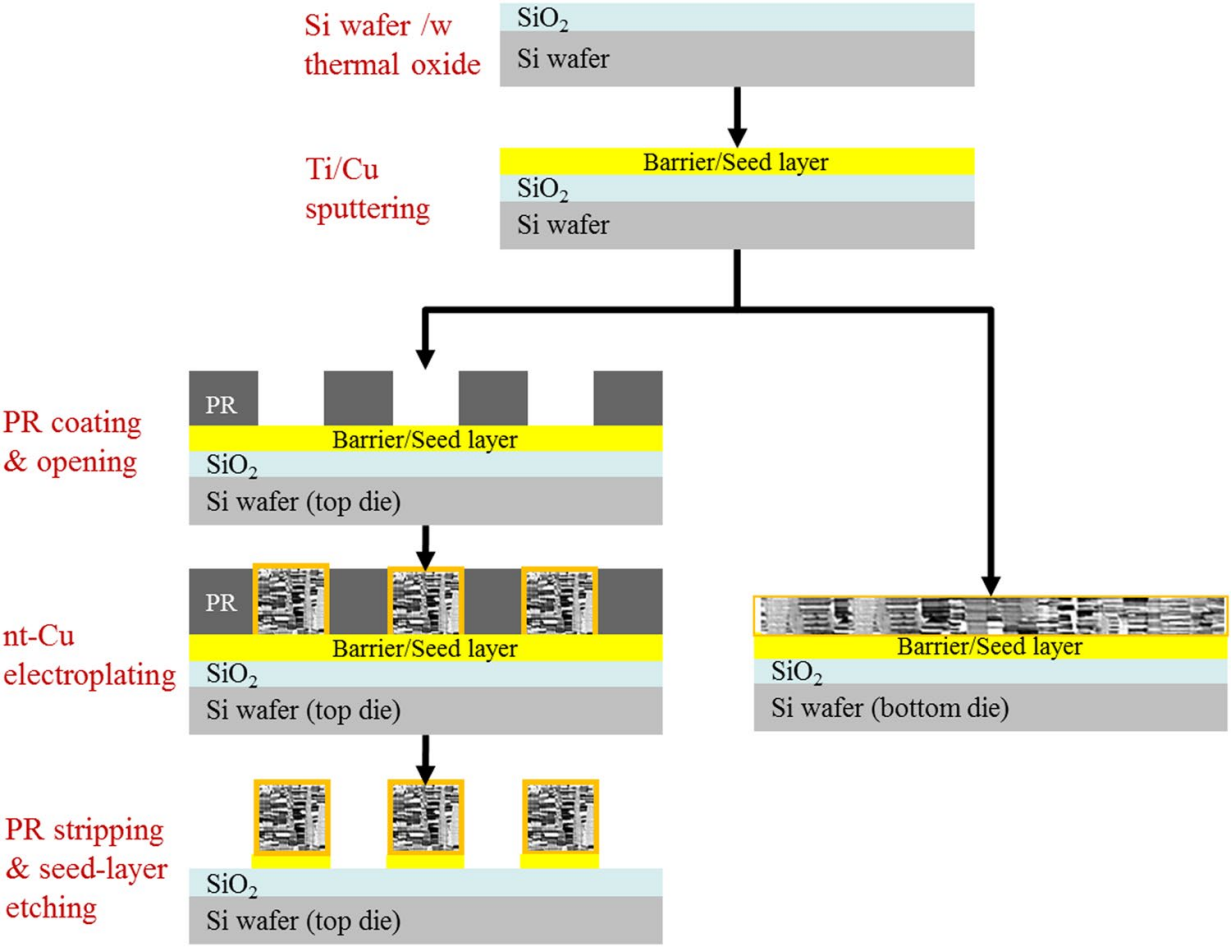
Schematic drawing of top and bottom die fabrication procedures.

**Figure 2:**
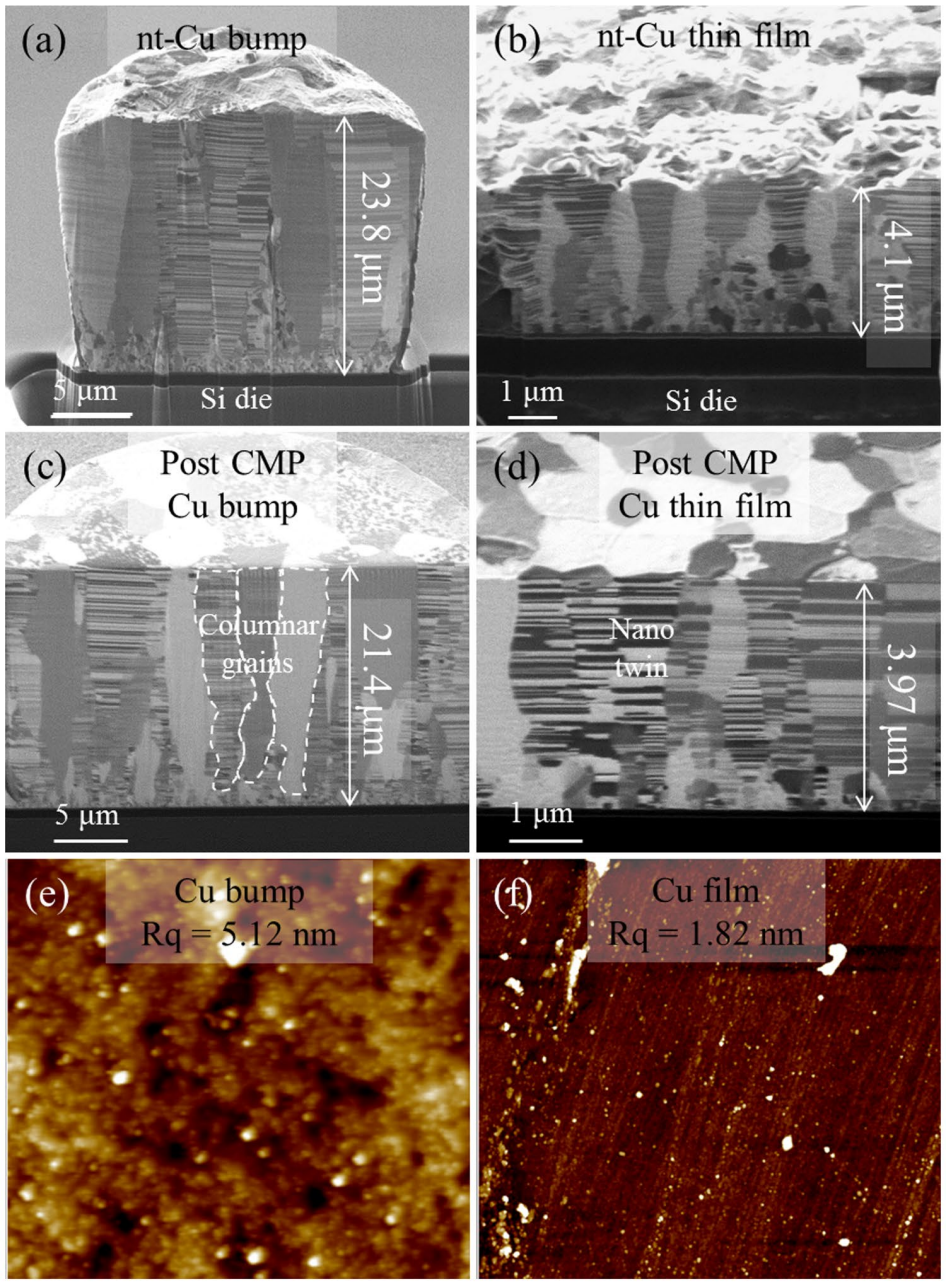
Cross-sectional SEM/FIB images of electroplated (**a**) nt-Cu bump, (**b**) nt-Cu thin film; cross-sectional SEM/FIB images of post-CMP (**c**) nt-Cu bump, (**d**) nt-Cu thin film; and AFM scanning results for (**e**) nt-Cu bump, (**f**) nt-Cu thin film.

Figure 1 illustrates the fabrication procedures of the test dies used in this study. In the first step, thermal oxidation was applied to produce a thin silicon oxide layer on the surface of Si wafers. Then, a 100 nm thick Ti diffusion barrier and 200 nm thick Cu seed-layer were sputtered on oxidized wafers by physical vapor deposition (PVD) process. Subsequently, the processed wafers were subjected to two different process steps for top and bottom die fabrication. For the top die, photolithography and electroplating were applied to form a nt-Cu pillar bump array. For the bottom die, only an electroplating process was performed to form a nt-Cu structured thin film. Figure 2a,b show the as-deposited microstructure of the pillar bump and thin film with nt-Cu, respectively. A texture of columnar grains with lamella nanotwins can be observed. Owing to the rough surface condition of Cu, the surfaces of the Cu pillar bump and thin film were flattened via chemical-mechanical-polishing (CMP) process. Figure 2c,d reveal the post-CMP Cu surface conditions. Figure 2e,f display the AFM scanning results. The measured root mean square roughness values (Rq) are 5.12 nm and 1.82 nm for the nt-Cu bumps and films, respectively. Before the bonding experiments, wet etching was performed to remove organic contaminants and the oxide layer. The test dies were rinsed with deionized water, followed by a short immersion in a mixed solution of citric acid and deionized water (in the ratio 133 g/100 ml) at 25 °C for about 30 s. Then, they were rinsed again with deionized water and dried by N_2_ purging before bonding.

### Cu-to-Cu direct bonding under a temperature gradient

**Figure 3:**
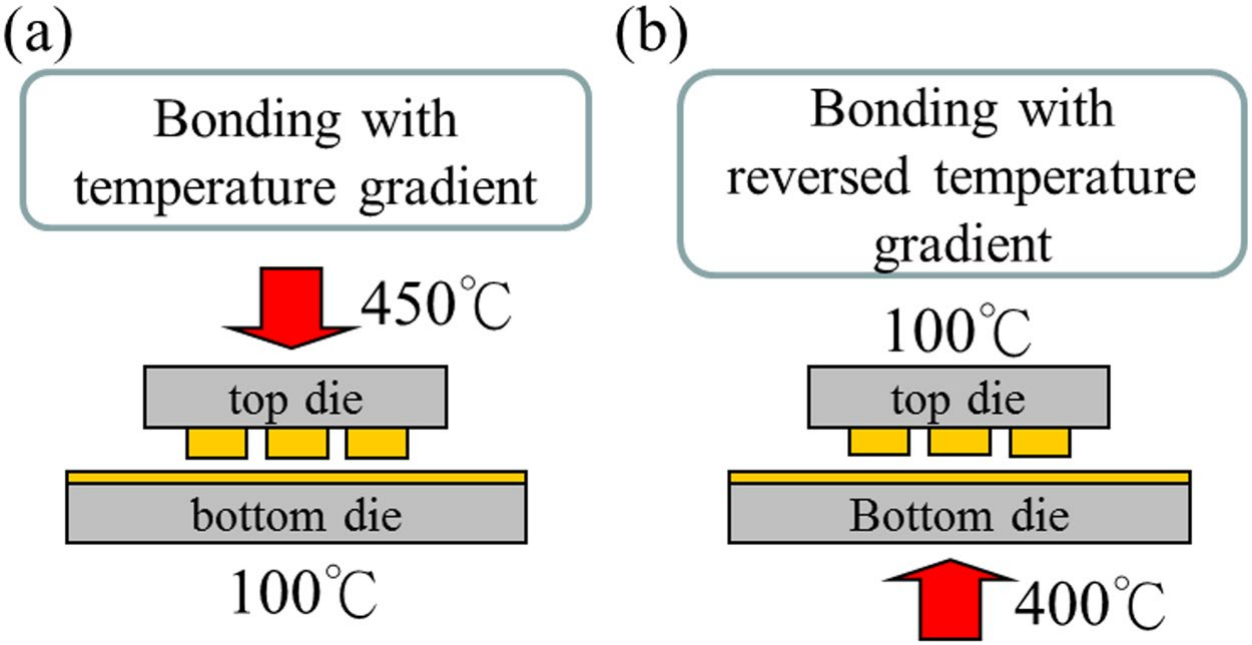
Schematic drawing of the bonding process with different temperature gradients. (**a**) Hot end (450 °C) applied to the top die, and (**b**) hot end (400 °C) applied to the bottom die.

Thermal compression bonding (bonding pressure = 162 MPa) was performed in a N_2_ purging atmosphere with a temperature gradient between the top and bottom dies, as shown in Fig. 3a,b. The schematic Fig. 3a depicts that the temperature gradient was between 450 °C (top die) and 100 °C (bottom die). However, the gradient is reversed in Fig. 3b, where the top die was at 100 °C and the bottom die at 400 °C. The reason for studying the reverse temperature gradient will be given later.

**Figure 4:**
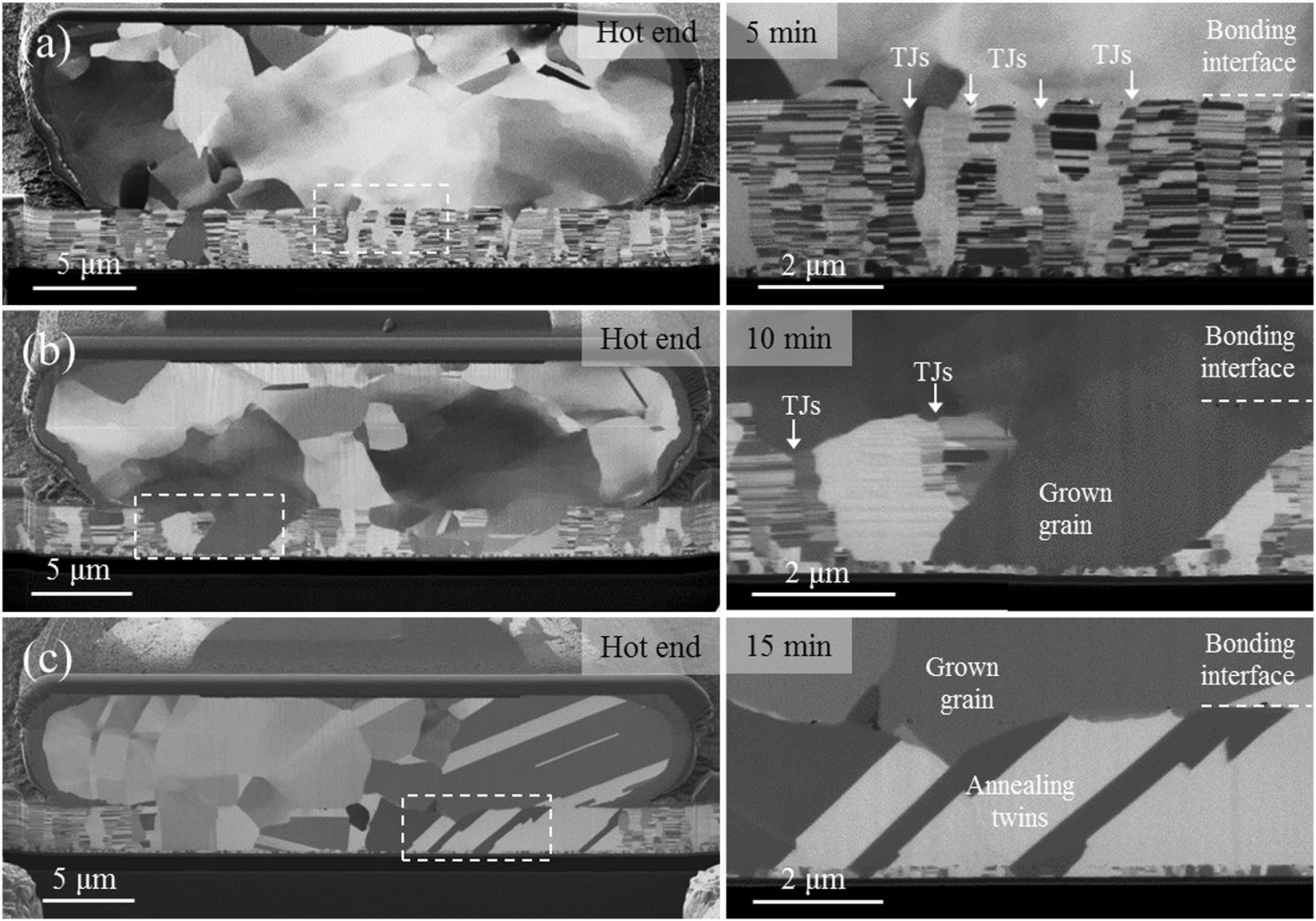
Variation in grain growth phenomena with bonding time for a temperature gradient between 450 °C at the top die and 100 °C at the bottom die: (**a**) 5 min; the nt-Cu in the bump has transformed to randomly oriented large grains, (**b**) 10 min; grain growth occurred through some of the large grains into the nt-Cu in the thin film, and (**c**) 15 min; grain growth with annealing twins occurred in most of the thin film. The areas corresponding to the dashed rectangular boxes in (**a**) to (**c**) are enlarged in the right-hand side images.

Figure 4 shows the cross-sectional focused ion beam (FIB) images of the bonded microstructure under different bonding time lengths of 5, 10, and 15 min for the temperature gradient depicted in Fig. 3a. Despite the fact that Cu oxidation cannot be prevented during bonding without vacuum, Fig. 4 shows well-bonded interfaces with a few voids. Importantly, the bonding time is only 5 min (short). Figure 4a reveals that grains evolved from the pillar bump. This is because high temperature and plastic deformation can trigger the recrystallization process in the pillar bump, but not in the thin film. We speculate that the pillar bump should store sufficient strain energy and dislocations for the nucleation and growth of strain-free grains during recrystallization^19^.

**Figure 5:**
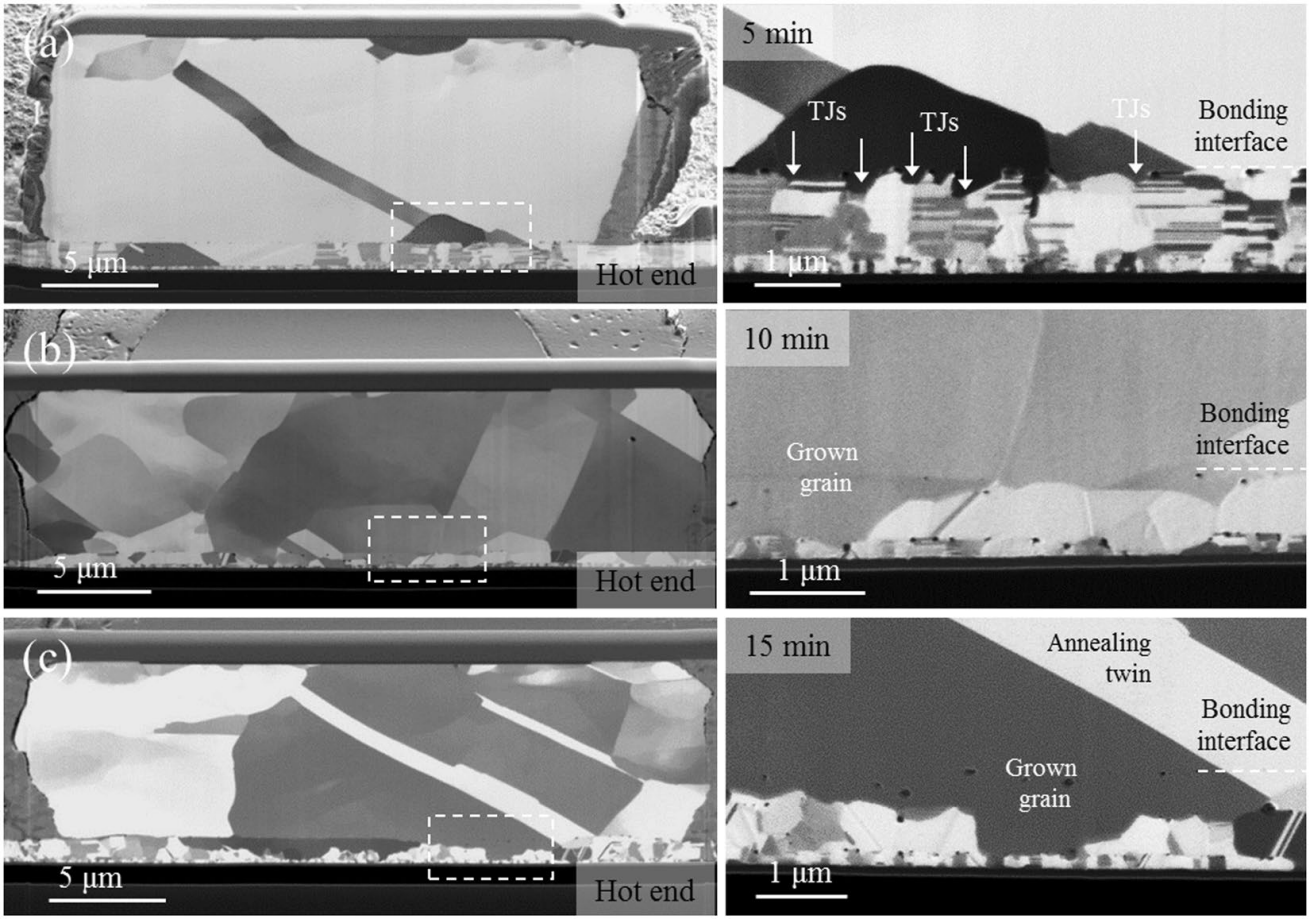
Reversed thermal bonding experiments by reversing the temperature gradient to 100 °C at the top die and 400 °C at the bottom die for bonding times of (**a**) 5 min; the nt-Cu in the bump has transformed to randomly oriented large grains, (**b**) 10 min; grain growth has occurred as some of the large grains grow into the nt-Cu thin film, and (**c**) 15 min; grain growth with annealing twins has occurred. The areas corresponding to the dashed rectangular boxes in (**a**) to (**c**) are enlarged in the right-hand side images.

Yet, no recrystallization occurred in the thin film. One possible reason is that its temperature was lower than the bump during compression bonding, as shown in Fig. 3a. Therefore, we carried out experiments under a reversed temperature gradient, as depicted in Fig. 3b, so that the thin film had a higher temperature. The outcome was shown to be the same, and is independent on the temperature gradient. Figure 5 shows the FIB images of the bonded interfaces under the reversed temperature gradient for varying bonding times. They have similar grain growth patterns, as shown in Fig. 4.

**Figure 6:**
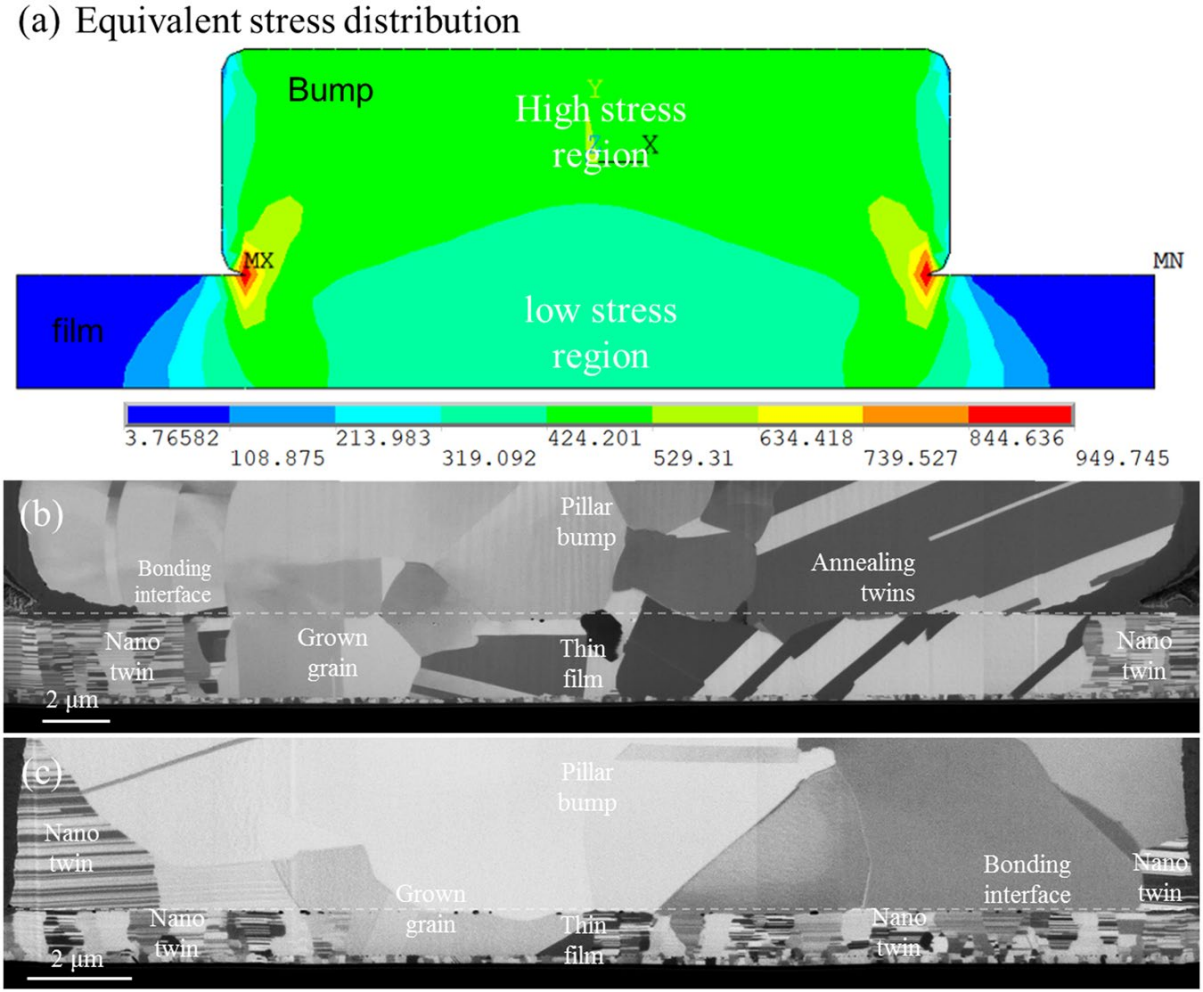
(**a**) Finite element analysis results of Cu-Cu direct bonding under stress and temperature gradient show that a stress potential exists in the bonding structure, and grain growth phenomena varied with the bonding stress at a temperature gradient between 450 °C at the top die and 100 °C at the bottom die for (**b**) 162 MPa and (**c**) 81 MPa bonding stress conditions.

Alternatively, the other possible reason may be uneven stress distribution during bonding. To verify this assumption, we performed a finite-element simulation with the bonding condition depicted in Fig. 3a. The finite element model in Fig. 6a reveals that a localized high-stress region occurs in the pillar bump, while a low-stress state simultaneously exists in the thin film right below the pillar bump. The pillar bump possesses higher stress than the Cu film, which could enhance the atomic diffusion for recrystallization, resulting in subsequent grain growth. On the other hand, the thin film, which is under a relatively low stress condition, is more stable against recrystallization. As a result, there is a significant difference in grain growth evolution between the bump and the thin film.

For a further investigation of the stress-assisted grain evolution, we made a comparative study between the 162 MPa and 81 MPa bonding stress conditions. For the stress condition at 162 MPa, most of the nt-structure along the bonding interface has been transformed into a few randomly oriented grains or grains with annealing twins (Fig. 6b). For the bonding stress condition of 82 MPa, the nt-grains in the upper pillar bump were transformed into a large grains and some upper grains in the pillar bump grew into the lower thin film (Fig. 6c). It is similar to the high-stress condition. However, many nt-grains on the thin film side can still be observed, unlike the results observed for the case of 162 MPa. Thus, we believe that the lower stress state in the thin film reduced the progress of grain evolution.

Upon further annealing, grain growth across the bonding interface started from the pillar bump and proceeded into the thin film. For this, direct bonding must be performed first. Therefore, we discuss the mechanism of bonding before reporting the grain growth across the bonded interface.

### Cu-to-Cu direct bonding mechanism

**Figure 7:**
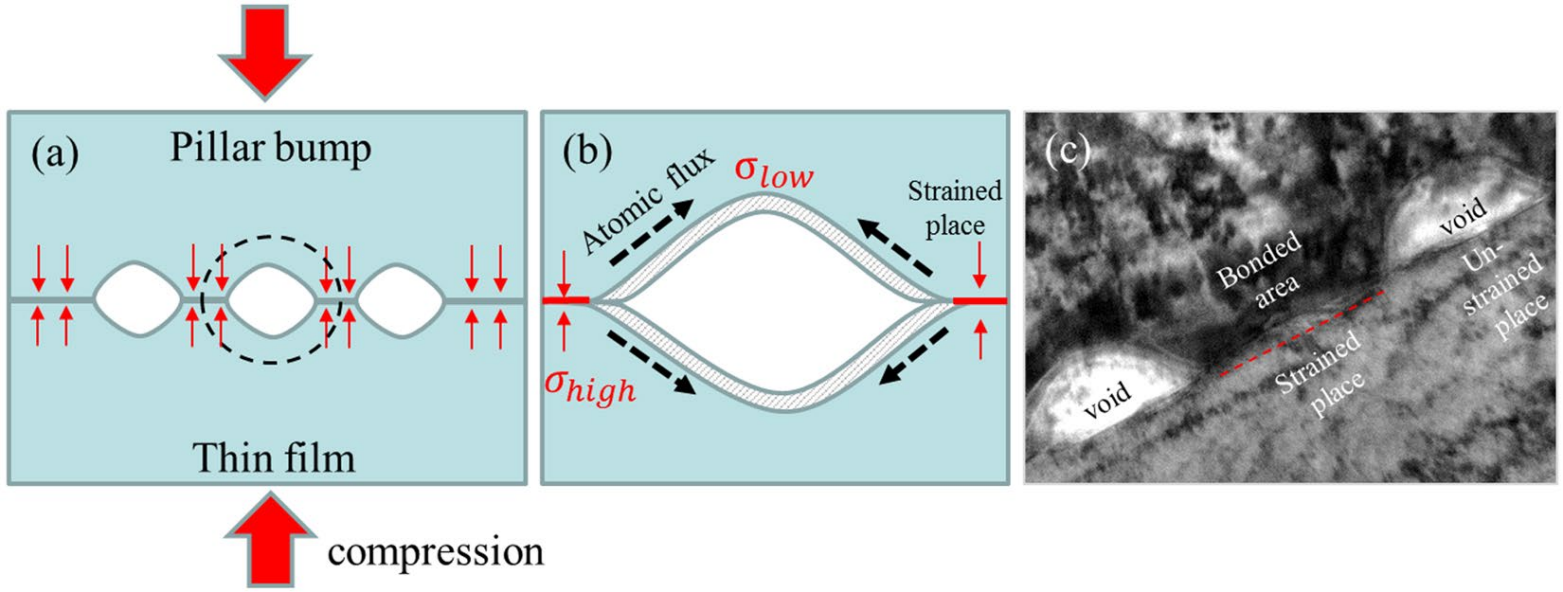
Schematic drawings of the metal-to-metal direct bonding mechanism. (**a**) Less perfect surfaces contact during the compression process, (**b**) atoms diffuse from regions of high stress (where two parts touch under compression) to those of lower stress (untouched under compression) along the interface, and (**c**) TEM image of the bonding interface showing the bonded (strained) and unbonded (unstrained) areas.

During thermal compression, stress-induced surface diffusion (creep) simultaneously occurred at the bonding interface. The surface diffusion-induced creep is similar to the model of Nabarro–Herring creep due to lattice diffusion and Coble creep by grain boundary diffusion^20,21^. In these creep models, the driving force for atomic flux is the stress potential gradient^22^. Hence, atoms or vacancies can migrate either within the grains or along the grain boundaries. In the present case, atomic diffusion occurs along the bonding interface. Under compression, the stress potential gradient occurs between the contacted regions and the non-contacted regions along the interface, as depicted by the schematic diagrams in Fig. 7a. This induces creep by surface diffusion to migrate atoms from the strained region to the unstrained (or void) region, as shown in Fig. 7b. This creep would in turn produce new atomic bonds of Cu-to-Cu across the interfaces. Figure 7c shows the result of the bonded region, including the contacted and non-contacted areas along the bonding interface.

### Bonding interface characterization

**Figure 8:**
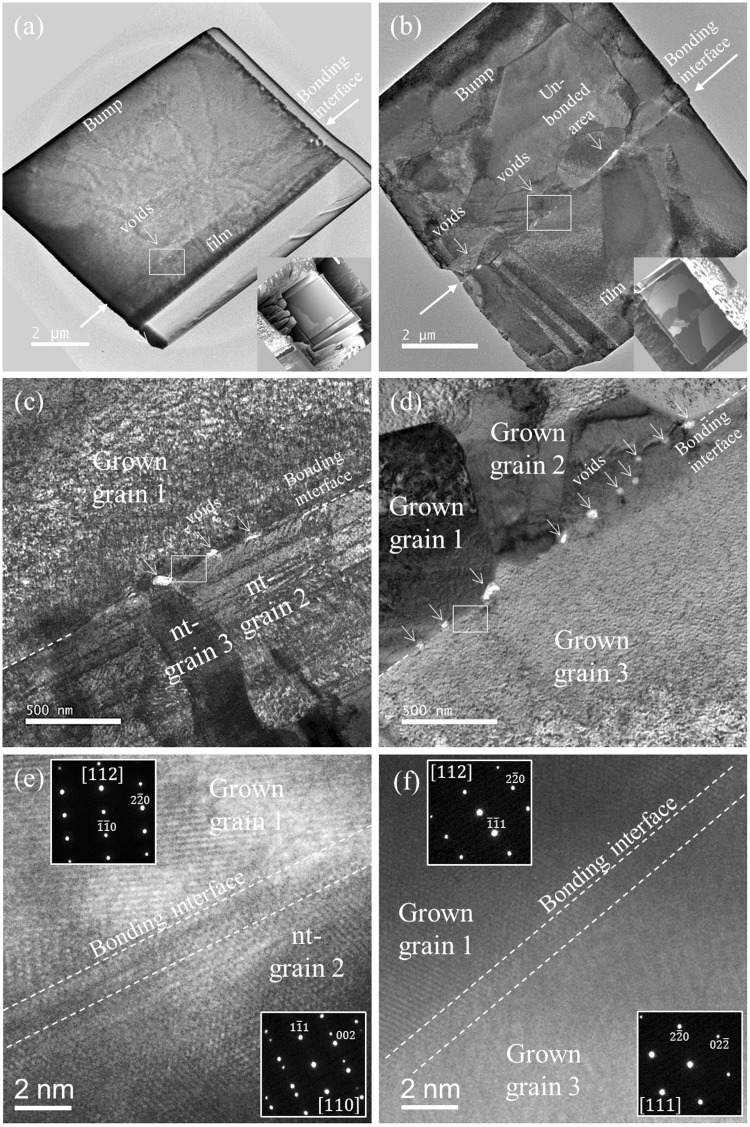
Characterization of the bonding interface between the bonded nt-Cu and bonded polycrystalline Cu: cross-sectional TEM images of the (**a**) bonded nt-Cu joint and (**b**) bonded polycrystalline Cu joint; the grain growth behavior and bonding interface condition for (**c**) bonded nt-Cu joint and (**d**) polycrystalline Cu joint; and the cross-sectional HRTEM images of the bonded interface and corresponding diffraction patterns for (**e**) bonded nt-Cu joint and (**f**) bonded polycrystalline Cu joint.

To validate the aforementioned unique grain growth behavior, a comparative study of nt-Cu and polycrystalline Cu was conducted. Both nt-Cu-to-nt-Cu and polycrystalline Cu-to-polycrystalline Cu were bonded at the temperature gradient between 450 °C and 100 °C for 15 min. Then, the bonded samples were subjected to the mechanical cross-sectioning process for microstructure observations. By comparing the cross-sectional images shown in Fig. 8a,b, different bonding scenarios were identified. It was found that the bonded nt-Cu has fewer voids at the bonding interface (Fig. 8a). In contrast, a region not bonded and numerous voids were found in the bonded polycrystalline Cu (Fig. 8b).

For a detailed structural analysis, two different morphologies were observed. Figure 8c shows the columnar grains in the pillar bump that have already agglomerated into a large size grain, which connected with the lower de-twined columnar grains in the thin film. At the bonding interface, very few voids can be found. For the bonded polycrystalline Cu, non nt-grains can be joined together, but several small voids (smaller than 100 nm) were found along the bonding interface (Fig. 8d). In order to characterize the bonded interface between the grains, high-resolution transmission electron microscopy (TEM) images (Fig. 8e,f) and the corresponding diffraction patterns were obtained. For both the bonded structures, no amorphous layer or discontinuous interface was observed, as indicated by the white dashed line. The zone axes in adjacent grains for bonded nt-Cu are [112] and [110]. For bonded polycrystalline Cu, the zone axes for the joined grains are [112] and [111].

For bonding of (111) nt-Cu bumps to (111) nt-Cu films, although the roughness values for the nt-Cu surfaces are larger than that of polycrystalline Cu surface, the average void size/area for the nt-Cu joints is smaller than that of the polycrystalline Cu joints. Using the TEM images in Fig. 8(a–d), we measured the area of interfacial voids, and the measured average void area for the nt-Cu joints is 2600 nm^2^, whereas it is 5500 nm^2^ for the polycrystalline Cu joints. There were 5 voids in the nt-Cu joint and 12 in the polycrystalline Cu joint. It was reported that the surface diffusion of Cu on (111) planes is faster by 3–4 orders in magnitude than other major planes^17,18^. Therefore, the (111) surface facilitates the diffusion of Cu atoms and then voids in the bonding interface can be filled by the Cu atoms. In addition, the nanotwins may be beneficial to reduce interfacial voids. It is reported that the nanotwins may serve as vacancy sinks to hinder the formation of Kirkendall voids during metallurgical reactions of Sn and Cu^23,24^. This is because there were many inherent twins in the electroplated Cu and the defects may be able to absorb vacancies. A statistical study on the void distribution needs to be performed in the future.

### Grain growth across the bonding interface from triple junctions

For grain growth across the bonded interface, we found that it initiates at the triple junctions (TJs) of grain boundaries, as shown in Fig. 4a. As the bonding time is increased to 10 min, well-grown grains are found at the TJs along the bonding interface in Fig. 4b. Some of the grains in the thin film merged to form a long, inclined grain with boundaries approaching the bottom seed-layer. For bonding time increased up to 15 min, annealing twins are observed in Fig. 4c, where straight-sided crystals have lattices arranged in a symmetrical manner. In Fig. 4a–c, the microstructure within the rectangular area of the dashed line has been enlarged in the images on the right-hand side. Similar grain growth is observed in Fig. 5.

**Figure 9:**
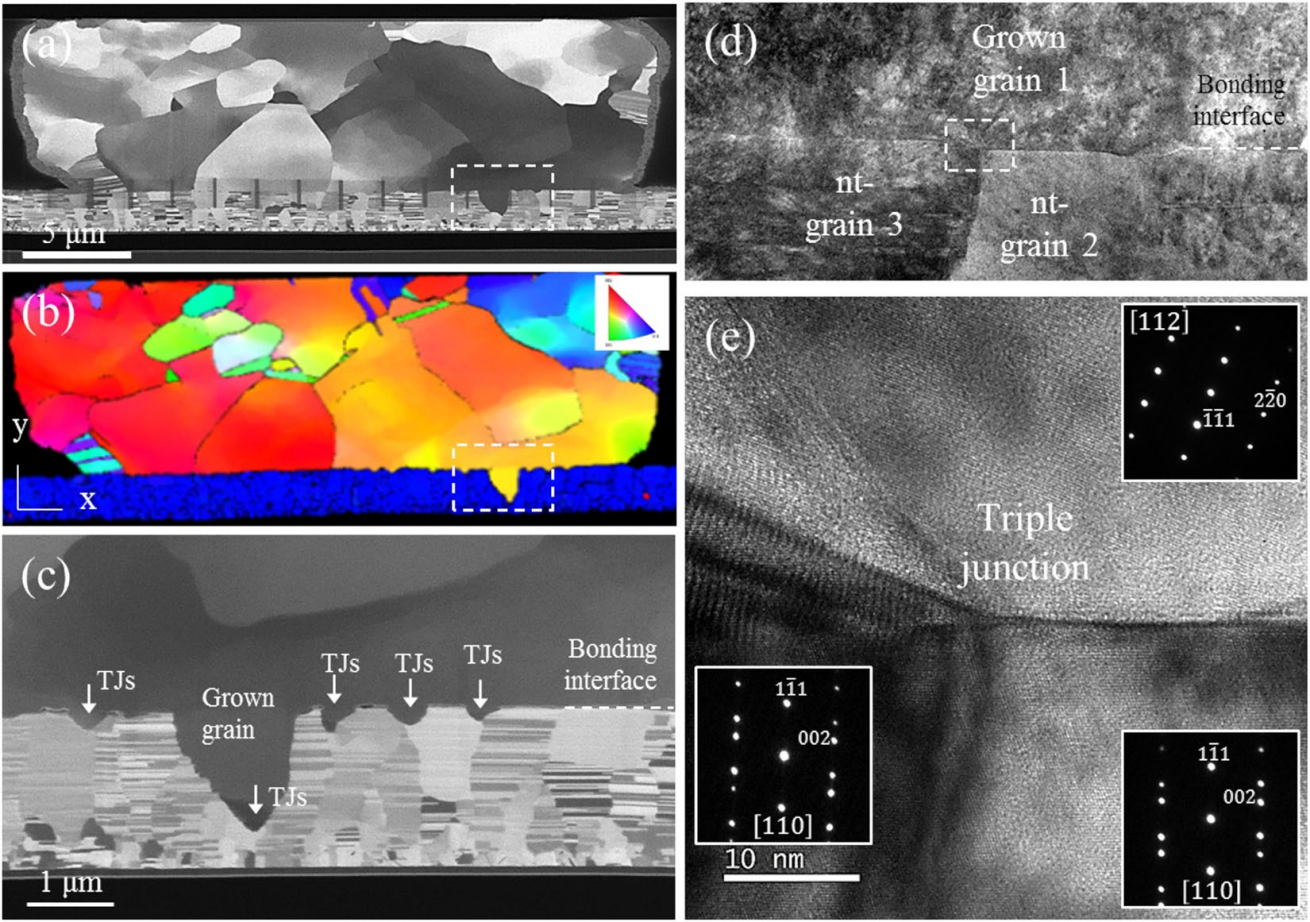
Observation of anisotropic grain growth behavior under the bonding condition of 400 °C at the top die and 100 °C at the bottom die for 20 min. (**a**) Cross-sectional view of the bonded joint, (**b**) grain orientation (IPF-Y) and boundaries, (**c**) grain growth initiated from the points of triple junctions. (**d**) TEM image of the triple junction at the bonding interface, and (**e**) cross-sectional HRTEM image corresponding to the dashed rectangular box in (**d**) showing the atomic arrangement in the region of the triple junction boundary and the corresponding diffraction patterns in the insets.

To study the grain growth behavior across the interface, we ensured a temperature gradient by maintaining the top die at 400 °C and the bottom die at 100 °C. Note that grain growth is quite rapid under the 450 °C/100 °C temperature gradient condition, so we lowered the bonding temperature to decrease the grain evolution. Figure 9a shows a cross-sectional FIB image of a well-bonded interface, and Fig. 9b an electron backscattered diffraction (EBSD) image of the grain orientations and boundaries. As indicated by the dashed squares in Fig. 9a,b, grain growth occurs from the upper to the lower side of the joint. In Fig. 9c, the FIB image of higher magnification shows that grain growth is initiated at the TJs of grain boundaries. We investigated the bonding interface based on the TJs through TEM observations. Figure 9d shows that the grains of the TJ consist of an upper grown grain and two lower nanotwinned columnar grains. Along the boundaries of the TJ, the atomic arrangement is disordered and loose (Fig. 9e); it differs from the dense packing of a face-centered cubic arrangement.

## Discussion

We analyze herein the mechanism of boundary movement and energy change. In (111)-oriented nt-Cu, having columnar grains, each grain consists of parallel twin lamellae with a high density coherent twin boundaries (CTBs). All the columnar grains have a common tilt axis, so all the grain boundaries are tilt-type grain boundaries. Besides, the tilt-type columnar grain boundaries (CGBs) contain a high density of TJs (where CTBs meet CGBs). It is reported that the TJs have a significant influence on grain growth due to the energetic or accelerating effect^25^. In addition, high-angle tilt-type boundaries (HATBs) have been confirmed to have greater mobility than low-angle tilt-type boundaries^26^. Therefore, CGBs with HATBs and a high density of TJs would enhance the recrystallization process^27^. An analysis to explain why the bonding interface movement is preferentially initiated at the points of TJs and extends along CGBs with HATBs is presented.

**Figure 10:**
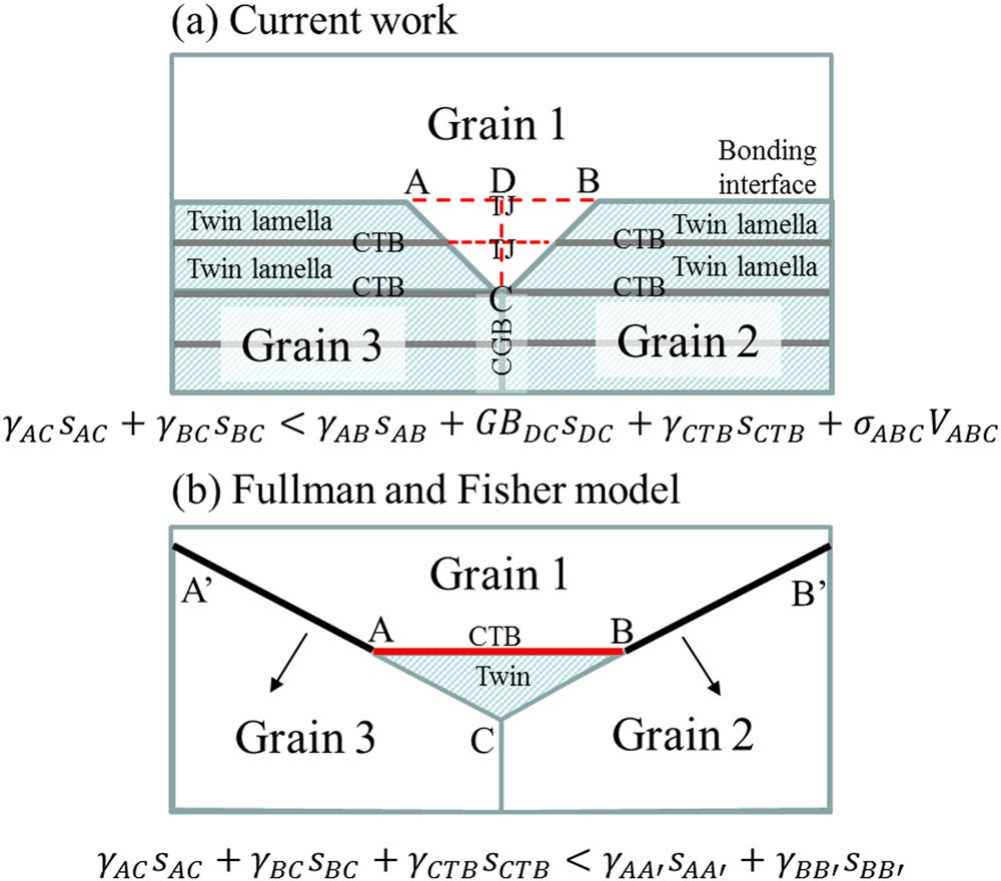
(**a**) Proposed anisotropic grain growth model for nt-Cu, and (**b**) the grain growth model for annealing twin formation, as reported by Fullman and Fisher.

In view of energy reduction, the recrystallization process resulting in the formation of new strain-free grains will attempt to minimize the grain boundary energy, twin energy, and strain energy involved. This is why while the upper grains grew into the lower grains in the thin film, as shown in Fig. 10a, the twin lamellae and their CTBs were consumed gradually. We express the energy change as

1 $${\gamma }_{AC}{s}_{AC}+{\gamma }_{BC}{s}_{BC} < {\gamma }_{AB}{s}_{AB}+G{B}_{DC}{s}_{DC}+{\gamma }_{CTB}{s}_{CTB}+{\sigma }_{ABC}{V}_{ABC}$$

where *γ* and *s* represent the interfacial free energy and area of the boundaries, respectively. The GB term corresponds to the grain boundary energy. The subscripts AB and DC refer to the grain boundaries before grain growth, and AC and BD refer to the new grain boundaries formed after grain growth. In addition, CTB indicates a coherent twin boundary. Moreover, the strain energy and volume are denoted by *σ* and *V*, respectively. If the inequality of the above equation is satisfied, then grain growth in the vertical direction can occur.

For comparison with the model of Fullman and Fisher^28^, annealing twin formation near the TJ during grain growth is a mechanism to reduce the free energy, as shown in Fig. 10b. The corresponding inequality can be written as

2 $${\gamma }_{AC}{s}_{AC}+{\gamma }_{BC}{s}_{BC}+{\gamma }_{CTB}{s}_{CTB} < {\gamma }_{AA{\rm{^{\prime} }}}{s}_{AA{\rm{^{\prime} }}}+{\gamma }_{BB{\rm{^{\prime} }}}{s}_{BB{\rm{^{\prime} }}}$$

However, it should be noted that our case is opposite to that of Fullman and Fisher. Their objective is to form a twin, whereas ours is to eliminate it. Nevertheless, both cases must lead to a reduction in the total energy. Furthermore, our case involves strain energy besides twin energy and grain boundary energy. Indeed, further studies on the kinetics of growth will be needed.

## Conclusions

In summary, vacuum-free Cu-to-Cu direct bonding using (111)-oriented nt-Cu has been achieved through thermal compression bonding for 5 min under a temperature gradient. To investigate the recrystallization occurring across the bonding interface, experiments with different bonding times and temperature gradients were performed. A surface diffusion creep-assisted bonding mechanism has been proposed to account for the observed direct bonding.

## Methods

### nt-Cu test using top/bottom dies

In this study, highly (111)-oriented nt-Cu was electroplated on the top and bottom dies. The dimensions of the test sample are 5 mm × 5 mm for the top die and 20 mm × 20 mm for the bottom die. The pillar bump array on the top die is 30 μm in diameter and 22 μm to 24 μm in height. The thickness of the thin film in the bottom die ranges from 2 μm to 4 μm. The bonding structure is designed to be amenable to the chip-to-wafer bonding die stacking structure. By this bonding structure, Cu-to-Cu direct bonding can be applied without an alignment process; it can be performed with a simple tool.

### Chemical-mechanical planarization

The liquid solution used in this study is the commercial high-removal rate chemical mechanical slurry TSV-C1015-02 (Cabot Microelectronics). It contains 0.3 wt.% colloidal silica as abrasive grits. The average grit size is around 70 nm in diameter, contains 3 wt.% of oxidant, hydrogen peroxide (H_2_O_2_), is added while polishing. The pH of the slurry is within the range of 3 to 4. The removal rate is around 800 nm/min to 1000 nm/min, depending on the pressure and plate-rotation speed. The slurry also contains an inhibitor to prevent the undesired removal of the concave surface, achieving global planarization and sub-nanoscale roughness on a finished surface. The measured root mean square roughness values (Rq) are 5.12 nm and 1.82 nm for the nt-Cu bumps and films, respectively. However, the measured Rq values are 2.53 nm and 0.69 nm for the polycrystalline Cu bumps and films, respectively.

### Pre-cleaning of Cu surface

Wet etching was applied to remove the organic contaminants and oxide layer before bonding. The test dies were rinsed with deionized water, followed by a short immersion in a mixed solution of citric acid and deionized water (133 g/100 ml) at 25 °C for about 30 s. Then, they were rinsed again with deionized water and dried using N_2_ purging before bonding.

### Direct bonding with temperature gradient

Thermal compression bonding was applied in a N_2_ purging atmosphere with a temperature gradient between top and bottom dies. For grain growth study, different bonding times of 5, 10, and 15 min were considered for the temperature gradient between 450 °C (top die) and 100 °C (bottom die). To determine whether the grain growth behavior is affected by the direction of the temperature gradient, we reversed the temperature gradient so that the top die was at 100 °C and the bottom die at 400 °C during the thermal compression process for the same bonding times (5, 10, and 15 min). In addition, for the observation of anisotropic grain growth behavior at the bonding interface, we lowered the temperature gradient by keeping the top die at 400 °C and the bottom die at 100 °C for 20 min.

### Examination of bonded interfaces

A FIB was employed to observe the bonded Cu-Cu interface and grain growth behavior. Electron backscattered diffraction was performed by a JSM-7800F PRIME field-emission scanning electron microscope with an EBSD detector Nordlys Max3. Aztec EBSD software was employed to analyze the orientation maps and crystallographic textures based on the Kikuchi patterns. The microstructures of the bonded interfaces were examined with a JEOL- JEM-F200 scanning transmission electron microscope. The TEM examinations were performed at 200 kV, with a point-to-point resolution of 0.23 nm and a lattice resolution of 0.14 nm. The surface roughness values of the Cu films were measured using scanning probe microscopy (Veeco Dimension 3100).

### Finite element simulation

Finite element analysis was carried out to simulate the thermomechanical behavior of the Cu to Cu direct bonding structure. Ansys software was adopted for the simulation, and Four-noded 182 Element was applied in the two-dimensional model. The total number of nodes and elements in this model were 59,431 and 57,928, respectively.
